# P30 A baseline evaluation of intermittent IV vancomycin management in the acute hospital setting following introduction of an electronic prescribing system (HEPMA)

**DOI:** 10.1093/jacamr/dlag102.036

**Published:** 2026-06-26

**Authors:** Rachael Rodger

**Affiliations:** Royal Alexandra Hospital, Paisley, NHS Greater Glasgow & Clyde, UK

## Abstract

**Background:**

In NHS Greater Glasgow and Clyde (NHSGGC) IV vancomycin management has been an ongoing focus of quality improvement (QI)^1-3^. Guidelines, dose calculators and specific prescribing, administration and monitoring (PAM) charts have been developed to improve management. In 2021, introduction of electronic prescribing (HEPMA) resulted in a change from exclusively paper to a combination of paper and electronic prescription/administration processes. Electronically intermittent IV vancomycin is prescribed ‘as required’ with no dose or frequency specified and paper PAM charts are used to document prescription/administration and therapeutic drug monitoring (TDM) specifics^4^. This change prompted the need to establish a new baseline understanding of intermittent IV vancomycin management and identify areas for targeted QI.

**Objectives:**

To collect baseline data to understand the current management of IV vancomycin at the Royal Alexandra Hospital (RAH) following introduction of HEPMA and identify areas for targeted QI.

**Methods:**

A data collection tool (Microsoft Forms) and HEPMA reports were used (March 2023- Feb 2024) to identify RAH adult inpatients prescribed intermittent IV vancomycin. Electronic prescriptions and paper PAM charts were reviewed prospectively, data collated using Excel, areas for improvement identified and QI targets set.

**Results:**

In total 98 adult patients (53% male), mean age 63 years from medical (41%), surgical (50%) and elderly care (9%) wards prescribed intermittent IV vancomycin were reviewed. The majority of IV vancomycin treatment durations were between 2-13 days (84%), with a small proportion less than 48 h (10%) or more than 14 days (6%). In line with pre-HEPMA data, the standard of dose calculations and prescribing/administration documentation on PAM charts were high. Loading and maintenance doses regimens were in line with guidelines in 97% and 96% of patients, respectively and the proportion of doses missed (3.9%) remained within target (Figure 1). A number of areas for targeted QI were identified and QI targets set (Figure 2): Delayed doses, 58% of loading (*n*=79) and 75% of first maintenance (*n*=79) doses were given on time (within 1 h of prescription). Overall, 75% of all IV vancomycin doses (*n*=843) were administered on time, compared to 83% pre-HEPMA. Only 44% of loading (*n*=79) and 59% of maintenance (*n*=764) dose administrations were documented on HEPMA compounded by the fact not all NHSGGC inpatient wards have HEPMA. Post HEPMA the proportion of maintenance doses (*n*=764) prescribed in the middle of the night (midnight-6am), a target of ongoing QI, reduced slightly to 18% compared to 22% (pre-HEPMA). Only 58% of TDM samples (*n*=188) were in line with guidelines. The main reasons for inappropriate samples were wrong sample time (37%), dose and/or sample time not accurately recorded (29%) or patient not at steady state (27%).

**Conclusions:**

Following introduction of HEPMA the standard of IV vancomycin dose and frequency calculations, PAM chart documentation and missed doses, remained high. Areas identified for targeted QI included: dose delays, late night prescribing, TDM and documentation of dose administrations on HEPMA. Setting QI targets and using a continuous QI approach moving forward will support optimization of IV vancomycin management and patient safety in NHSGGC.

**Figure 1.**
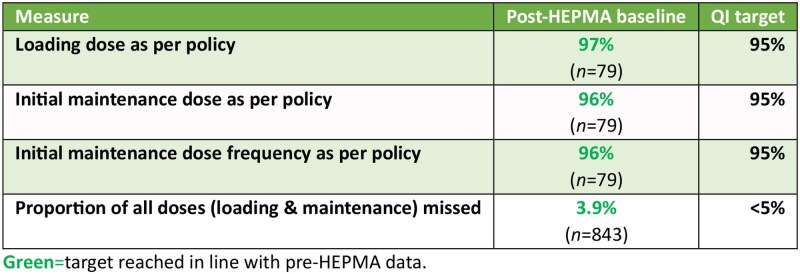
Post-HEPMA intermittent IV vancomycin measures at baseline as per NHSGGC Prescribing Administration & Monitoring (PAM) charts.

**Figure 2.**
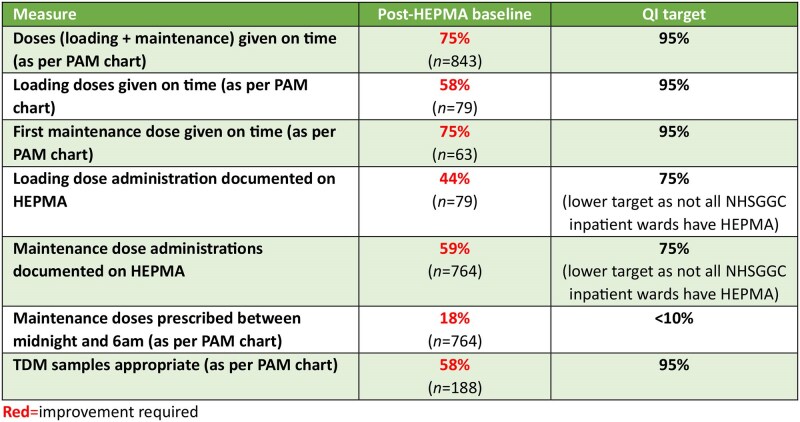
Areas of IV vancomycin management identified as requiring improvement as per NHSGGC Prescribing Administration & Monitoring (PAM) charts and HEPMA with QI targets.
